# *Campylobacter jejuni* Surface-Bound Protease HtrA, but Not the Secreted Protease nor Protease in Shed Membrane Vesicles, Disrupts Epithelial Cell-to-Cell Junctions

**DOI:** 10.3390/cells13030224

**Published:** 2024-01-25

**Authors:** Irshad Sharafutdinov, Nicole Tegtmeyer, Manfred Rohde, Annelie Olofsson, Zia ur Rehman, Anna Arnqvist, Steffen Backert

**Affiliations:** 1 Department of Biology, Division of Microbiology, Friedrich-Alexander-Universität Erlangen-Nürnberg, Staudtstr. 5, D-91058 Erlangen, Germany; 2 Central Facility for Microscopy, Helmholtz Centre for Infection Research, Inhoffenstraße 7, D-38124 Braunschweig, Germany; 3 Department of Medical Biochemistry and Biophysics, Umeå University, SE-901 87 Umeå, Sweden

**Keywords:** *Campylobacter*, serine protease HtrA, tight junctions, occludin, E-cadherin

## Abstract

Fundamental functions of the intestinal epithelium include the digestion of food, absorption of nutrients, and its ability to act as the first barrier against intruding microbes. *Campylobacter jejuni* is a major zoonotic pathogen accounting for a substantial portion of bacterial foodborne illnesses. The germ colonizes the intestines of birds and is mainly transmitted to humans through the consumption of contaminated poultry meat. In the human gastrointestinal tract, the bacterium triggers campylobacteriosis that can progress to serious secondary disorders, including reactive arthritis, inflammatory bowel disease and Guillain–Barré syndrome. We recently discovered that *C. jejuni* serine protease HtrA disrupts intestinal epithelial barrier functions via cleavage of the tight and adherens junction components occludin, claudin-8 and E-cadherin. However, it is unknown whether epithelial damage is mediated by the secreted soluble enzyme, by HtrA contained in shed outer-membrane vesicles (OMVs) or by another mechanism that has yet to be identified. In the present study, we investigated whether soluble recombinant HtrA and/or purified OMVs induce junctional damage to polarized intestinal epithelial cells compared to live *C. jejuni* bacteria. By using electron and confocal immunofluorescence microscopy, we show that HtrA-expressing *C. jejuni* bacteria trigger efficient junctional cell damage, but not soluble purified HtrA or HtrA-containing OMVs, not even at high concentrations far exceeding physiological levels. Instead, we found that only bacteria with active protein biosynthesis effectively cleave junctional proteins, which is followed by paracellular transmigration of *C. jejuni* through the epithelial cell layer. These findings shed new light on the pathogenic activities of HtrA and virulence strategies of *C. jejuni*.

## 1. Introduction

The intact intestinal epithelium plays important roles in both absorption of nutrients and protection of adjacent tissues from the entry of microbiota that inhabit the lumen [1]. Epithelial cells form a monolayer lining, in which cell connectivity is mediated via a specialized apical–junctional complex [2,3]. Various transmembrane proteins, including occludin, tricellulin, claudins, junctional adhesion molecules (JAMs) and E-cadherin, establish and maintain cell-to-cell interactions and extracellular barrier functions. In the cytoplasm, the scaffolding proteins zonula occludens (ZO) and β-catenin connect the junctional transmembrane proteins with cytoskeletal components and mediate intracellular signalling [4,5]. Due to these important host cell functions, many pathogens have evolved strategies for entering the epithelial layer through interaction with the components of the apical–junctional complex [6,7]. The affected junctional components can trigger deregulation of the cell polarity network, permit pathogen invasion into deeper tissues and enable bacterial spread [8,9,10]. For example, well-known microbes such as pathogenic *Escherichia coli*, *Helicobacter pylori* and *Shigella flexneri* exploit the apical-junctional complex to efficiently infect the host [7]. In addition, the gastrointestinal pathogen *Campylobacter jejuni* targets multiple proteins of the apical–junctional complex such as occludin, claudin-8 and E-cadherin, the disruption of which facilitates paracellular transmigration of this bacterium across the intestinal epithelium and provokes various pathologies [11].

*C. jejuni* represents a major foodborne bacterium that causes inflammatory infections of the gut associated with diarrhoea, called campylobacteriosis [12]. This pathogen typically colonizes the lower intestine of birds and various mammals as a commensal. The optimal growth temperature of thermophilic *C. jejuni* species is well adapted to the normal body temperature of birds (about 39–42 °C) [13]. The principal infection route of *C. jejuni* to humans occurs through the ingestion of contaminated poultry meat, raw milk or water [12,13]. Various *C. jejuni* pathogenicity-associated mechanisms were reported, such as bacterial motility, chemotaxis, biofilm formation, cell binding and invasion, intracellular survival and epithelial translocation [14,15]. The symptoms of the resulting campylobacteriosis disease include abdominal pain, diarrhoea with blood and fever [16,17]. The clinical outcome of campylobacteriosis can be acute for some infected persons, lasting up to one or two weeks, but is usually self-limiting in most individuals. However, some infected patients develop more severe post-infectious autoimmune diseases affecting the nervous system and joints, such as reactive arthritis as well as the Guillian–Barré and Miller Fisher syndromes [18,19,20]. In addition, *C. jejuni* infection has been linked to inflammatory bowel disease (IBD) [21,22]. IBD is a term which describes inflammatory ulcerative colitis and Crohn’s disease; these conditions appear to originate from autoimmune responses against the normal microbiota [23]. Interestingly, *C. jejuni* infection of infant mice revealed specific shifts in the composition of the gut microbiota [24]. Furthermore, it was demonstrated that microbiota dysbiosis in the intestine can arise due to infection with various pathogenic microbes, which can trigger chronic inflammatory responses [25]. In particular, the presence of *C. jejuni* and *E. coli* was implicated to accelerate IBD. In this regard, it was demonstrated in vitro that *C. jejuni* infection can trigger the translocation of commensal bacteria such as *E. coli* across polarized epithelial cell monolayers, which may explain the development of IBD [21,22,26].

Recent studies provided evidence that a major disease-associated factor of *C. jejuni* is the serine protease HtrA (high-temperature requirement A) [27,28,29]. HtrA assembles in the bacterial periplasm as active trimers that form higher oligomers such as hexamers and dodecamers [30] that protect the pathogen against various extracellular stresses [31,32]. Studies in the mouse model systems of *C. jejuni* infection revealed that *C. jejuni* HtrA intensifies campylobacteriosis symptoms by triggering pro-inflammatory reactions and apoptosis [33,34,35]. HtrA seems further to be involved in *C. jejuni* binding and invasion of intestinal epithelial cell lines in vitro by a yet unknown mechanism [31,36,37,38,39]. In addition, *C. jejuni* wild-type (wt) bacteria can rapidly transmigrate across polarized epithelial cells that were grown in transwell filter systems in vitro [37]. Bacterial transmigration is associated with cell-damaging effects associated with the disruption of junctional scaffold proteins, an effect that is strongly diminished by infections with ∆*htrA* deletion or protease-inactive *C. jejuni.* This suggests that *C. jejuni* traveling across the epithelium requires HtrA protease activity. In fact, *C. jejuni* HtrA can cleave *in vivo* and in vitro two tight junction proteins, claudin-8 and occludin [40,41], as well as the major tumour suppressor and adherens junction component E-cadherin [37,42]. These cleavage events compromise the epithelial barrier and allow *C. jejuni* to migrate via the paracellular route across the intestinal epithelium, which fosters the infection [11].

The above-described observations suggest that extracellular delivery of HtrA by *C. jejuni* is required for cleavage of junctional proteins, which is followed by intestinal epithelial barrier disruption [28]. In particular, two distinct pathways of HtrA secretion into the extracellular environment have been considered: either secretion as a soluble enzyme or in shed outer-membrane vesicles (OMVs). OMVs are generated by all Gram-negative bacteria, including *C. jejuni*, and form when the bacterial outer membrane buds outwards, followed by the release of membrane-surrounded vesicles containing soluble factors [43]. Proteomic analysis identified about 185 proteins in *C. jejuni* OMVs, such as periplasmic and outer membrane-associated proteins, including an array of virulence factors such as cytolethal distending toxin (CDT), adhesins such as MOMP (major outer membrane protein) and serine proteases (Cj0511, Cj1365c and HtrA), as well as lipooligosaccharides [44,45,46]. However, the secretion rates of *C. jejuni* OMVs over time are unknown. Alternatively, HtrA was found as a soluble enzyme in the bacterial culture supernatant, with the two *C. jejuni* model strains NTCT11168 and 81–176 yielding similarly high secretion rates of soluble HtrA. For example, strain 81–176 secreted about 6 × 10^12^ molecules per mL at two hours, which increased to 10 × 10^12^ molecules after eight hours of growth in liquid broth [47]. Thus, it was proposed that the above-described cleavage of junctional proteins and epithelial cell damage could be mediated by either secreted soluble HtrA, and/or by shed OMVs, or by HtrA anchored at the bacterial outer membrane. Here, we performed experiments to study the efficiency of soluble HtrA, of HtrA in purified OMVs and of bacterial outer membrane-bound HtrA to trigger epithelial cell disruption. We found that recombinant HtrA and OMVs barely caused any cell damage, even at very high concentrations. In contrast, metabolically active *C. jejuni* bacteria cleaved junctional proteins very fast and highly effectively disrupted the epithelial cell layer during infection. We propose a new molecular model to explain these surprising results.

## 2. Materials and Methods

### 2.1. Bacterial Strains and Cultivation

The *C. jejuni* isolate 81–176 wt, the isogenic Δ*htrA* deletion and complemented S197A point mutant strains were previously described [37,38]. These *C. jejuni* strains were cultivated using *Campylobacter* blood-free selective agar base plates supplemented by *Campylobacter* growth complement (Oxoid, Wesel, Germany). In case of antibiotic-resistant strains, kanamycin (20 μg/mL) was added to the growth medium. The bacteria were routinely grown for two days at 37 °C in anaerobic jars and under microaerobic conditions produced by CampyGen^TM^ gas packs from Oxoid and were harvested using sterile cotton swabs. For quantification of the bacterial cells, *C. jejuni* was resuspended in liquid Mueller–Hinton broth, and the optical density (OD) at 600 nm was measured in an Eppendorf spectrophotometer.

### 2.2. Purification of Recombinant C. jejuni HtrA (rHtrA)

Cloning of the *C. jejuni htrA* gene (corresponding to amino acids 17–472) was performed as described [37,42]. In brief, the *htrA* gene was amplified by PCR from genomic *C. jejuni* DNA without the predicted signal peptide (corresponding amino acids 1–16). The primers were ligated to *Bam*HI or *Xma*I restriction sites, respectively, to generate PCR fragments for cloning into the pGEX-6P-1 expression vector (GE Healthcare, Chicago, IL, USA). Expression of rHtrA was performed as a fusion with a GST (glutathione S-transferase) tag. Purification of HtrA-GST was reported previously [48].

### 2.3. Preparation of Outer Membrane Vesicles (OMVs)

Purification of *C. jejuni* OMVs was performed as described [49] with various minor adaptations that were previously reported for OMVs prepared from the related pathogen *H. pylori* [50]. Briefly, the *C. jejuni* strains were cultivated on agar plates as described above and then for about two days in Mueller–Hinton broth medium until OD of 0.5. OMVs were collected in Tris-HCl buffer (20 mM, pH 8.0). All further steps were performed at 4 °C. Two centrifugation steps followed at 8000× *g* for 30 min. The resulting supernatant was filtered via passage through a 0.22 µm cellulose acetate membrane and subjected to ultracentrifugation at 150,000× *g* for 3 h. The OMVs in the pellet were carefully resolved in 20 mM Tris-HCl buffer (pH 8.0) and further purified using a continuous 20–60% Histodenz gradient (Sigma-Aldrich, St. Louis, MO, USA) in 20 mM Tris-HCl buffer (pH 8.0) and 250 mM sucrose. The average size of the collected OMVs was measured via electron microscopy as described below. The protein content in the OMVs was quantified via standard Bradford assay.

### 2.4. Cell Culture and Infection Assays

Human intestinal epithelial Caco-2 cells (#HTB-37, ATCC, Manassas, VA, USA) were routinely cultured in 75 cm^2^ tissue culture flasks in Dulbecco’s Modified Eagle Medium (DMEM) supplemented with 10% FCS (both from Invitrogen, Waltham, MA, USA). MDCK (#CCL-34, ATCC) epithelial cells were grown in RPMI1640 medium containing 4 mM glutamine (Invitrogen) and 10% FCS. Culturing of all cell lines was performed in a humidified atmosphere at 37 °C and 5% CO_2_. For infection experiments, epithelial cells were seeded in 12–well plates and grown for 14 days to allow proper cell polarization, then were starved overnight in plain culture medium without FCS. Then, the cells were infected with *C. jejuni* using multiplicities of infection (MOI) of 20 or treated with either OMVs or recombinant HtrA (rHtrA) at concentrations and periods of time as indicated in the text or figure legends. Afterwards, E-cadherin cleavage detection was performed as described [51] or the infected cells were washed two times with pre-warmed PBS buffer, followed by preparation for microscopy, as described below.

### 2.5. Transwell System and Transepithelial Electrical Resistance (TER) Measurement

Polarized Caco-2 and MDCK cells were grown in 12-well plates with 0.33 cm^2^ culture inserts of 3 μm pore size (Merck Millipore, Darmstadt, Germany) using media as described above. Polarized Caco-2 cell monolayers were differentiated for 14 days [40]. Establishment of proper tight junctions during growth in the Transwell system was examined by measuring TER every two days until maximal values of 350–400 Ohm × cm^2^ were achieved. TER values were determined by the Electrical Resistance System (ERS, Merck Millipore). *C. jejuni* infection was performed in a time course of up to 16 h, as indicated in the text. In some experiments, we added chloramphenicol (20 μg per mL) or α-HtrA antibodies (2 μg per mL) [37] in the apical chamber prior to infection. The number of transmigrated CFU (colony forming units) from the bottom chamber was calculated after growth of serial dilutions on Mueller–Hinton agar plates [52]. To distinguish *C. jejuni* wt and Δ*htrA* mutant cells, the agar plates were supplemented with the respective antibiotics.

### 2.6. Field Emission Scanning Electron Microscopy (FESEM)

Plate-grown *C. jejuni* strains were harvested and fixed in a fixation solution with 5% formaldehyde and 2% glutaraldehyde in cacodylate buffer (0.1 M cacodylate, 0.01 M CaCl_2_, 0.01 M MgCl_2_, 0.09 M sucrose, pH 6.9) for 1 h on ice. Fixation of uninfected MDCK or Caco-2 cells, or cells treated with *C. jejuni*, purified OMVs or rHtrA, as described in the text, was performed with 5% formaldehyde and 2% glutaraldehyde in HEPES buffer (0.1 M HEPES, 0.09 M sucrose, 0.01 M CaCl_2_, 0.01 M MgCl_2_, pH 6.9) for 1 h. After several washing steps with cacodylate/HEPES buffer and TE buffer (20 mM Tris, 1 mM EDTA, pH 6.9), samples were dehydrated in serial dilutions of acetone (10, 30, 50, 70, 90 and 100%) on ice for 15 min each step. The samples were then allowed to reach room temperature before another change of 100% acetone, after which the samples were subjected to critical-point drying with liquid CO_2_ (CPD030, Bal-Tec, Balzers, Liechtenstein or CPD300, Leica, Wetzlar, Germany). The samples were finally covered with an approximately 10 nm thick gold/palladium film via sputter coating (SCD500, Bal-Tec, Florence-Graham, CA, USA) and examined in a field emission scanning electron microscope (Zeiss Merlin, Oberkochen, Germany) using an Everhart Thornley SE-detector and in-lens SE-detector in a 50:50 or 25:75 ratio at an acceleration voltage of 5.0 kV.

### 2.7. Transmission Electron Microscopy for Negatively Stained Samples

*C. jejuni* bacteria were negatively stained on a thin carbon support film (prepared on freshly cleaved mica). Samples were absorbed onto the carbon film by floating the carbon film on a drop of the samples. The film was then washed with TE buffer and subsequently negatively stained with 1% (*w/v*) aqueous uranyl acetate (pH 4.5) and blotted dry on a filter paper. After air drying, samples were examined via transmission electron microscopy (TEM) in a Zeiss TEM 910 (Oberkochen, Germany) at an acceleration voltage of 80 kV. Images were recorded digitally at calibrated magnifications with a Slow-Scan CCD-Camera (ProScan, 1024 × 1024, Scheuring, Germany) with ITEM-Software 2.1 (Olympus Soft Imaging Solutions, Münster, Germany).

### 2.8. Immunofluorescence Microscopy

Caco-2 and MDCK monolayers were grown on glass coverslips in 12-well plates as described above. Prior to treatment with *C. jejuni,* OMVs or rHtrA, the cells were washed with PBS and starved in plain DMEM without FCS and antibiotics [53,54]. Immunofluorescence staining and microscopy were performed as described previously, with minor modifications [55]. After treatment, the samples were washed with PBS and fixed with either 2% PFA (paraformaldehyde) or with ice-cold methanol for 15 min. For the staining of cell monolayers, samples were permeabilized with 0.25% Triton X-100. Labelling of cell junction proteins was performed using mouse α-E-cadherin (#610182, BD Biosciences, Heidelberg, Germany) or FITC-conjugated α-occludin (#331511, Invitrogen, Waltham, MA, USA). OMVs or rHtrA were stained using rabbit α-MOMP or α-HtrA antibodies [38], respectively. Goat α-mouse-TRITC (#T5393, Sigma-Aldrich) or α-rabbit-Alexa Fluor 633 (#A-21070, Invitrogen, Darmstadt, Germany) were used as secondary antibodies, and DAPI (4′-6-diamidino-2-phenylindole dihydrochloride; Invitrogen, Darmstadt, Germany) was used to counterstain nuclei. Next, the stained samples were analysed using the confocal laser scanning microscope Leica Stellaris 8 (Leica Microsystems, Wetzlar, Germany) at the Optical Imaging Centre Erlangen (OICE, Erlangen, Germany). Image acquisition was performed using the LAS AF computer software, version 4.4.0.24848 (Leica Microsystems). The cell junction integrity was analysed using micrographs of the FITC channel containing occludin staining. The occludin patterns were segmented into the regions of interest (ROI) in the ImageJ software, version 1.53c [56]. The relative fluorescence intensities of at least 50 ROIs per condition were measured and expressed as mean ± SD (standard deviation). The obtained fluorescence intensity values were analysed using the D’Agostino–Pearson test for normality followed by the one-way ANOVA test.

### 2.9. SDS-PAGE and Western Blotting

Cell lysates of non-infected or infected samples were harvested in hot 1x SDS-PAGE buffer. Standard 6–8% SDS-PAGE gels were used and blotted onto PVDF membranes. The blotted membranes were blocked with 5% skimmed milk in TBST buffer (25 mM Tris–HCl pH 7.4, 140 mM NaCl, 0.1% Tween-20) for 1 h at 20 °C. Afterwards, mouse α-E-cadherin (Santa Cruz Biotechnology, Dallas, TX, USA), α-HtrA [37] antibodies were added for 12 h at 4 °C. As secondary antibodies we utilized horseradish peroxidase-conjugated α-mouse immunoglobulins (Life Technologies, Darmstadt, Germany). Development was performed using the ECL Plus chemiluminescence kit (GE Healthcare) using standard protocols [57].

### 2.10. Casein Zymography

Bacterial cell pellets, purified OMVs or rHtrA were resuspended in PBS and mixed with Laemmli buffer. Further, samples were loaded onto 10% SDS-PAGE gels supplemented with 0.1% casein (Carl Roth, Karlsruhe, Germany). Separation was performed under non-reducing conditions. Renaturation of proteins within the gels was performed by washing in 2.5% Triton X-100 solution for 1 h at room temperature. Equilibration was performed overnight in the developing buffer (50 mM Tris-HCl, pH 7.4, 200 mM NaCl, 5 mM CaCl_2_, 0.02% Brij35) at 37 °C. Caseinolytic activity was assessed via detection on transparent bands, which indicated digestion of the casein substrate, after staining with 0.5% Coomassie Blue R250 as described [48].

### 2.11. Statistical Analyses

All experiments were performed in triplicate. The data were evaluated via two-tailed Mann–Whitney test, if not indicated otherwise, with GraphPad Prism 9 (Version 9.3.1). The obtained *p*-values *p* < 0.001 (***) were defined as statistically significant or ns—non-significant.

## 3. Results

### 3.1. Electron Microscopic Analysis of C. jejuni and OMV Production

**Figure 1:**
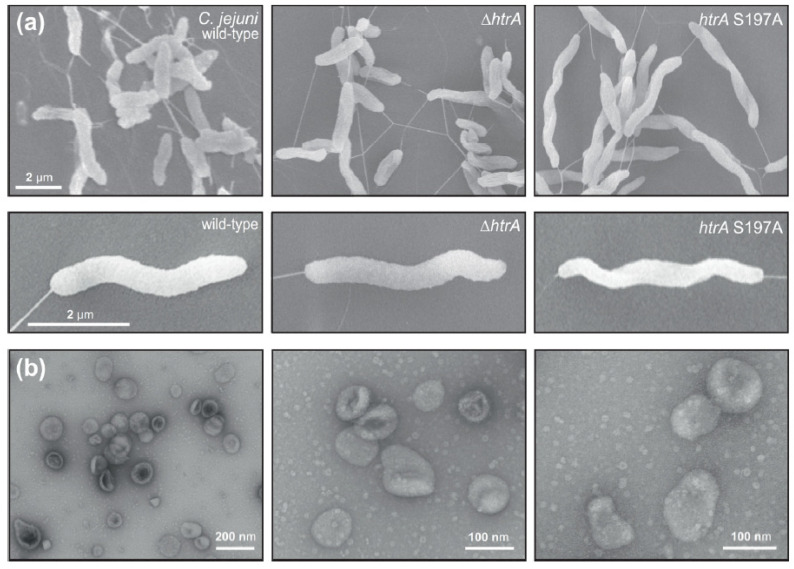
Electron microscopy of *C. jejuni* wild-type (wt) and *htrA* mutants utilized in this study. (**a**) Wt strain 81–176*,* isogenic Δ*htrA* mutant and *htrA* S197A point mutant revealed typical and similar cell morphologies. (**b**) Negative staining electron microscopy of purified OMVs from actively dividing wt *C. jejuni* confirmed the pureness of the isolated particles.

Serine protease HtrA plays an important role in the pathogenesis of many bacterial pathogens. In general, three major pathways of HtrA delivery onto host cells have been proposed, including (i) secretion of soluble HtrA, (ii) secretion of HtrA within OMVs, and (iii) presentation of HtrA anchored in the bacterial membrane [28]. We used the *C. jejuni* model strain 81–176 to study which of these proposed methods may account for most of the *C. jejuni*-induced epithelial cell damage. First, we examined the morphology of wild-type (wt) *C. jejuni,* an isogenic Δ*htrA* deletion mutant, and a strain with complemented *htrA* carrying a point mutation (serine to alanine) at position 197 (*htrA* S197A) that generated a proteolytically inactive enzyme, using scanning electron microscopy (FESEM). No significant phenotypical variations were detected between the *C. jejuni* wt and the corresponding *htrA* mutant strains (Figure 1a). In addition, all mutant strains exhibited intact bipolar flagella and were as motile as their wt counterpart. Using standard protocols, we biochemically purified OMVs from actively dividing *C. jejuni* strains harvested from liquid cultures at the exponential growth phase. The high purity of OMVs and absence of broken OMVs or cell debris were confirmed via negative staining with uranyl acetate and electron microscopy. In good agreement with previous *C. jejuni* OMV studies [44,45], the OMVs ranged between 20 nm and 200 nm in diameter (Figure 1b). The size and purity of the OMVs generated from various *C. jejuni* wt and mutant strains were similar.

### 3.2. Are OMVs the Primary Route for HtrA Delivery in C. jejuni–Host Interactions?

**Figure 2:**
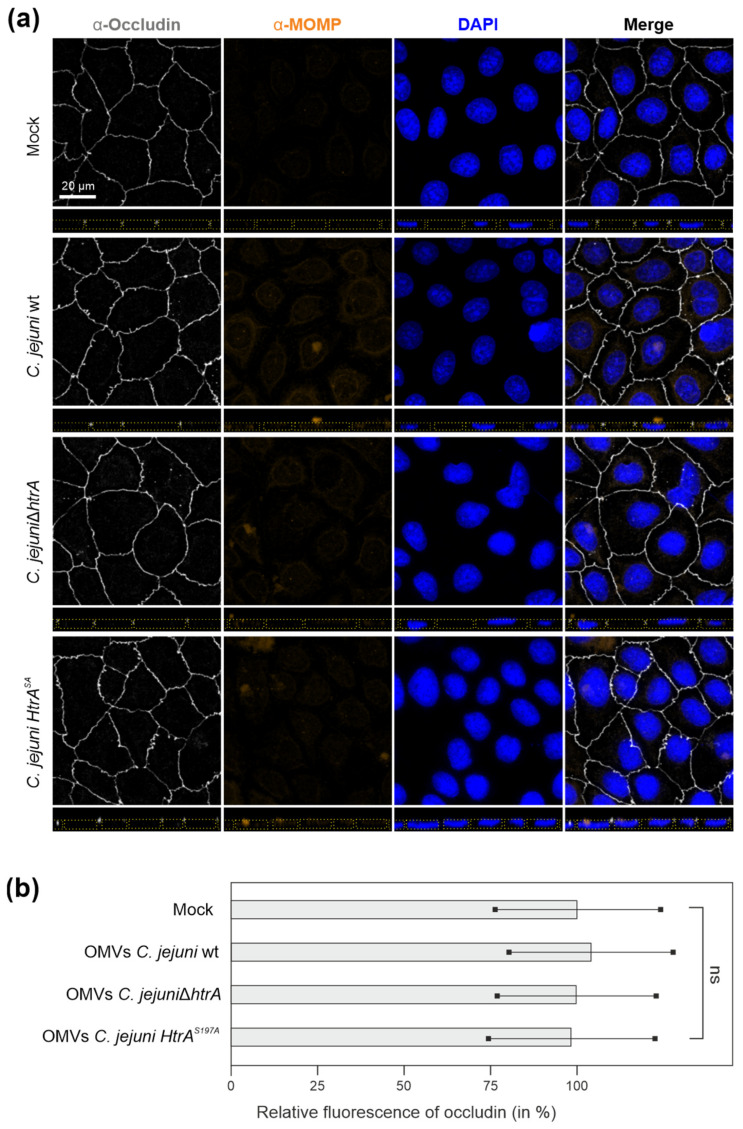
(**a**) Immunofluorescence microscopy of Caco-2 monolayers treated with *C. jejuni* OMVs. The cells were co-incubated for 12 h with 10 μg/mL OMVs purified from *C. jejuni* wt, or HtrA-inactive mutants Δ*htrA* or complemented *htrA* S197A. Caco-2 monolayers were fixed in ice-cold methanol and further stained to allow visualization of tight junctions (grey), OMVs (orange) and cell nuclei (blue) using α-occludin, α-MOMP and DAPI, respectively. Dotted yellow lines mark the cell peripheries in the z-stacks. (**b**) Integrity of Caco-2 cellular junctions after OMV treatment was assessed via measurement of the relative fluorescence intensity of occludin patterns obtained from the fluorescence microscopy images. One-way ANOVA test followed by Tukey’s multiple comparisons test revealed no significant difference between the groups; ns—not significant.

Previous studies indicated that HtrA present in secreted OMVs can promote *C. jejuni* invasion by mediating the cleavage of cellular junctions in intestinal epithelia [46]. The authors showed that 10 μg of purified *C. jejuni* OMVs resulted in proteolytic activity that nearly corresponded to that of 10 ng/mL trypsin [46]. On the other hand, *C. jejuni* is capable of secreting about 1 μg/mL of HtrA after 8 h growth, suggesting a major portion of secreted protein is being dissolved in the supernatant [47]. Therefore, we first aimed to study the role of OMVs in HtrA-dependent cleavage of tight junctions. For this purpose, accurately polarized Caco-2 cell monolayers, which were differentiated for about 14 days [46] and displayed TER values of 350–400 Ohm × cm^2^, were treated with 10 μg/mL of OMVs isolated from either *C. jejuni* wt, Δ*htrA* or *htrA* S197A mutants. The samples were immunostained for the tight junction component occludin, OMVs and cell nuclei using α-MOMP antibodies and DAPI, respectively, followed by analysis of the Caco-2 monolayers via fluorescence microscopy. Surprisingly, we did not observe any visible occludin damage in the tight junctions of Caco-2 cells even after 12 h of co-incubation with OMVs isolated from *C. jejuni* wt bacteria, which was similar to the control treatment with OMVs from HtrA mutant strains (Figure 2a). Quantification of relative fluorescence intensities from occludin-stained tight junctions revealed no significant difference between treated and mock control monolayers (Figure 2b).

### 3.3. Efficiency of C. jejuni OMVs and Soluble rHtrA in Cleavage of Tight Junctions

**Figure 3:**
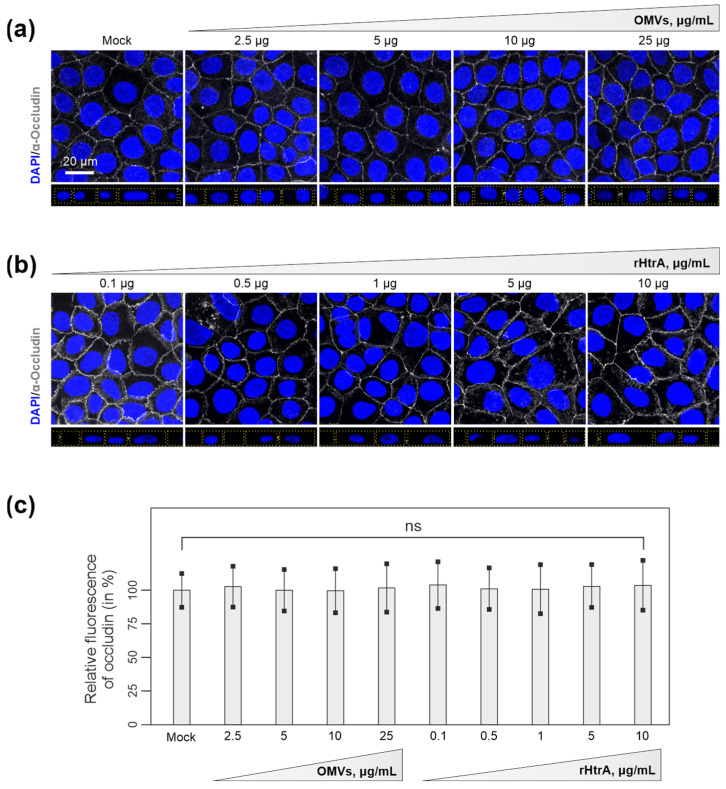
Immunofluorescence microscopy of Caco-2 monolayers after 6 h treatment with different concentrations of *C. jejuni* OMVs (2.5 μg/mL to 25 μg/mL) (**a**) or rHtrA (0.1 μg/mL to 10 μg/mL) (**b**). Caco-2 monolayers were fixed in 2% PFA and stained for tight junctions (grey) and cell nuclei (blue) using α-occludin and DAPI, respectively. Dotted yellow lines mark the cell peripheries in the z-stacks (**c**) Integrity of tight junctions in Caco-2 monolayers after treatment with OMVs or rHtrA was assessed as described above by measuring the relative fluorescence intensity of occludin-stained patterns in micrographs. One-way ANOVA with Tukey’s multiple comparison tests revealed no significant difference between the groups; ns—not significant.

**Figure 4:**
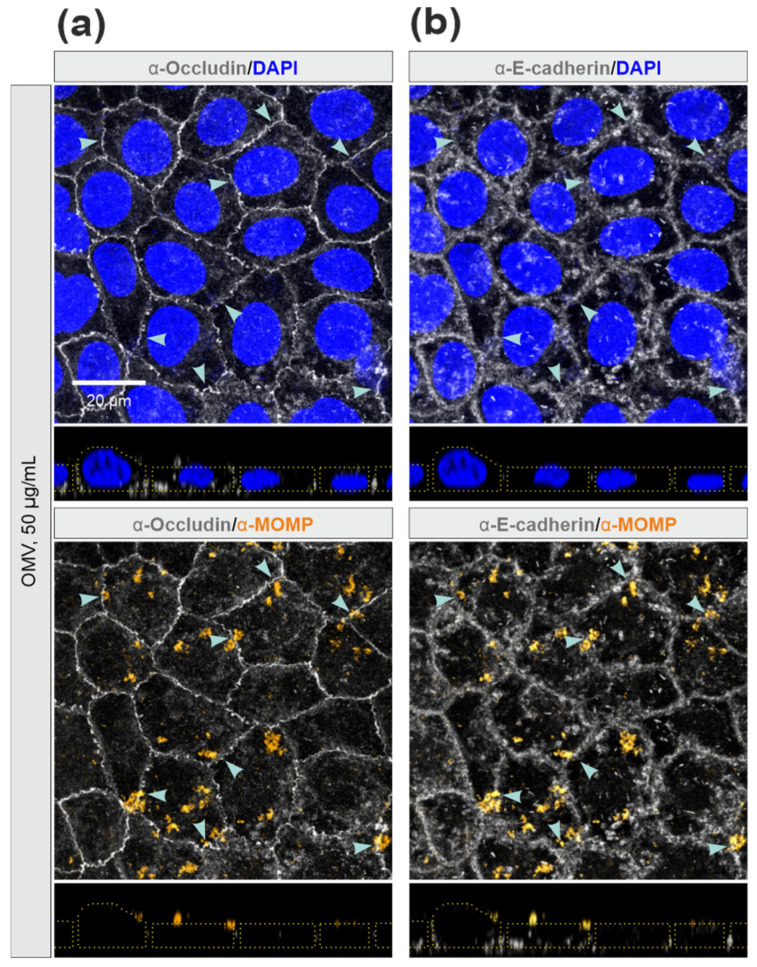
Immunofluorescence microscopy of Caco-2 monolayers after 6 h treatment with *C. jejuni* OMVs (50 μg/mL). Monolayers were fixed in 2% PFA followed by staining of OMVs (orange) with α-MOMP, and cellular junctions (grey) with either α-occludin (**a**) or α-E-cadherin (**b**). Cell nuclei were counterstained with DAPI (blue). Blue arrowheads indicate Caco-2 cellular junctions stained with occludin and E-cadherin in close proximity to the OMVs. Dotted yellow lines mark the cell peripheries in the z-stacks.

Next, we wanted to investigate whether disruption of cellular junctions can be achieved by higher concentrations of OMVs, or alternatively, by soluble rHtrA. To this end, we treated Caco-2 cell monolayers with increasing concentrations of OMVs from *C. jejuni* wt (2.5 μg/mL to 25 μg/mL) or rHtrA (0.1 μg/mL to 10 μg/mL). Remarkably, even treatment with the highest concentrations of *C. jejuni* wt OMVs or rHtrA did not result in a visible disruption of the tight junctions (Figure 3a,b). Analyses of occludin fluorescence intensities in the tight junctions revealed no significant differences between treated and control Caco-2 monolayers (Figure 3c). Finally, we treated Caco-2 monolayers with 50 μg/mL of *C. jejuni* wt OMVs. Along with the increased OMV concentration, we observed the characteristic OMV-stained spots similar to those observed in a previous work [53]. Again, no significant damage was observed in the junction proteins occludin and E-cadherin in close proximity to OMV-stained patterns (Figure 4a,b). OMVs were evenly spread throughout the cell surface, suggesting no specific accumulation of OMVs at cellular junctions. However, we observed a portion of OMVs that co-localized with E-cadherin outside of the junctions, though this was not indicative of protein digestion, since the junctional staining of E-cadherin in proximity to the OMVs was not affected. It was noted that at least 185 different proteins can be present within *C. jejuni* OMVs [58], and some of those could potentially interact with E-cadherin, which would result in their co-localization. This hypothesis should be addressed in future studies. Apparently, fluorescence microscopy did not indicate proteolytic cleavage of Caco-2 cellular junctions by either purified rHtrA or HtrA-containing *C. jejuni* OMVs in vitro. In contrast, bacterial infection with live *C. jejuni* has recently been shown to visibly damage epithelial monolayers, as was evidenced by fluorescence microscopy [26,40,41]. This suggested that other conditions during infection are involved in cellular proteolysis rather than soluble or OMV-associated HtrA.

### 3.4. C. jejuni Wt Infection of Epithelial Cells Results in Very Efficient Junctional Disruption

**Figure 5:**
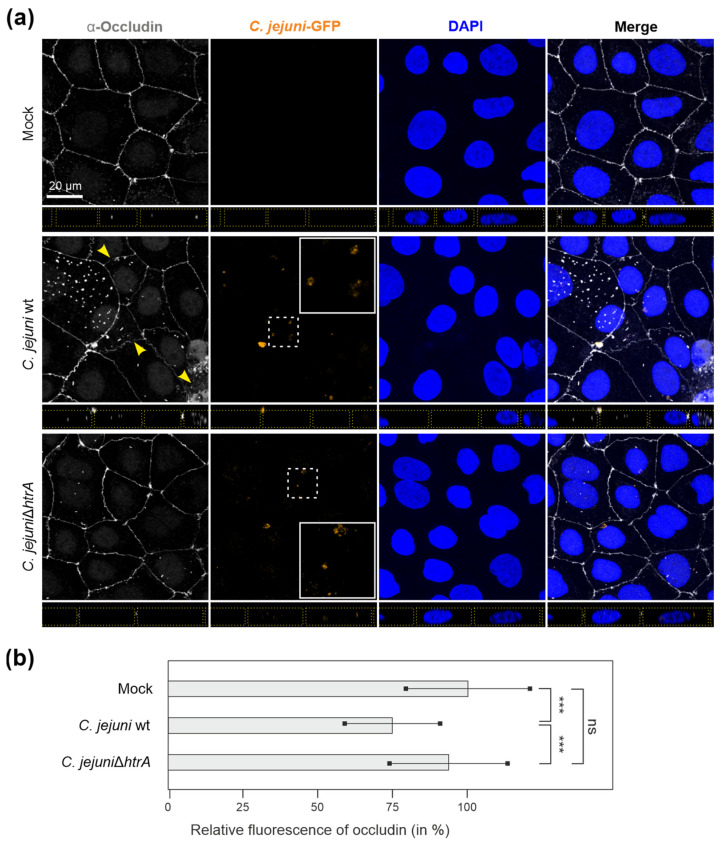
(**a**) Immunofluorescence microscopy of Caco-2 monolayers infected with GFP-tagged *C. jejuni* wt or Δ*htrA* mutant bacteria for 6 h. Fixed Caco-2 monolayers were immunostained using α-occludin (grey), and counterstained with DAPI (blue). Representative areas with *C. jejuni* bacteria (white dashed boxes) are enlarged (white solid boxes) for details. Yellow arrowheads indicate the areas with disrupted tight junctions. Dotted yellow lines mark the cell peripheries in the z-stacks. (**b**) Integrity of Caco-2 cellular junctions after infection was assessed via measurement of the relative fluorescence intensity of occludin patterns obtained from the fluorescence microscopy images. Differences between the groups were analysed via the one-way ANOVA test followed by the Tukey’s post hoc test; ***—*p* < 0.001; ns—not significant.

Previous studies showed that *C. jejuni* efficiently disrupts host cellular junctions during infection, with HtrA protease being the major bacterial factor maintaining the cleavage [37,40,41]. Indeed, fluorescence microscopy revealed alternated occludin distribution in Caco-2 monolayers upon infection with *C. jejuni* wt bacteria, but not with the isogenic Δ*htrA* mutant (Figure 5a). *C. jejuni* wt bacteria led to a partial loss of occludin from the tight junctions (Figure 5a, yellow arrows) along with reduced occludin mean fluorescence within tight junctions (Figure 5b).

**Figure 6:**
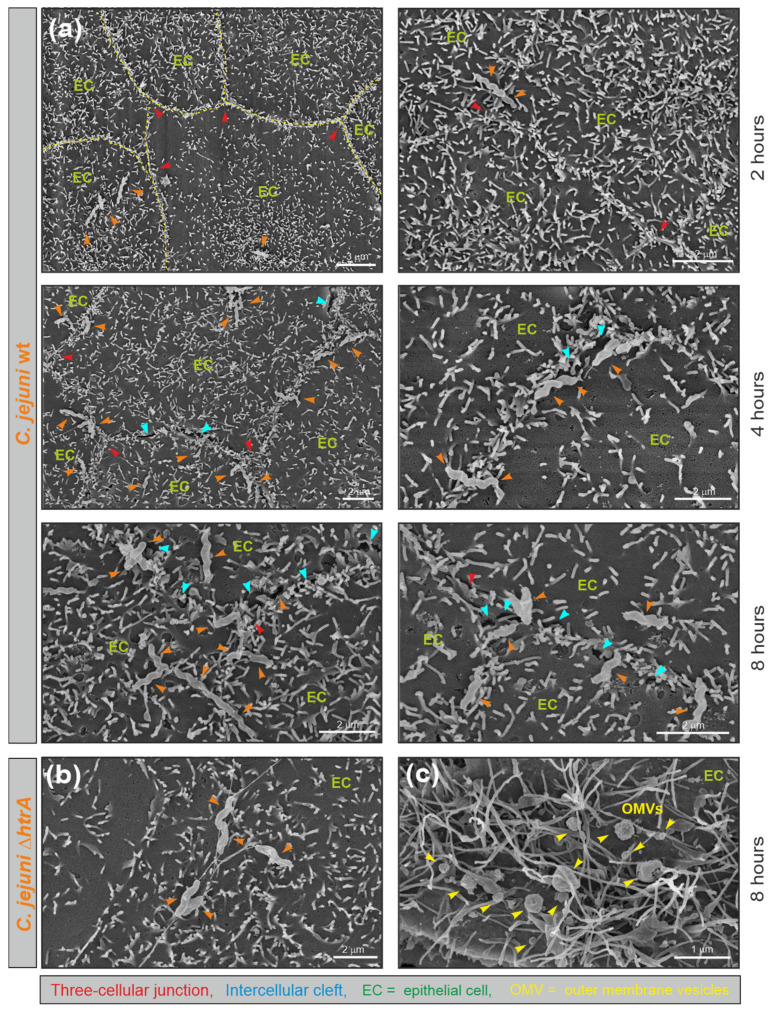
FESEM of polarized MDCK monolayers infected with *C. jejuni* wt and Δ*htrA* mutant or treated with wt OMVs. (**a**) MDCK cells were infected for 2 h, 4 h and 8 h, respectively. Top views of representative examples are shown. Various epithelial cells (EC) are shown. Cell junctions are visible as microvilli-dense areas. In the top left example, the junctions are labelled with yellow dashed lines. *C. jejuni* are marked with orange arrowheads, tricellular junctions with red arrowheads and induced intercellular clefts with blue arrowheads. (**b**) Infection with the Δ*htrA* mutant. (**c**) Co-incubation of cells with 50 μg/mL wt OMVs (yellow arrowheads).

For comparison, polarized Caco-2 cells and a second polarized cell line, MDCK, were infected with *C. jejuni* at a MOI of 20. Both cell lines showed similar results. FESEM of the cells revealed typical cell junctions and the presence of white-coloured microvilli as apical markers (Figure 6). These microvilli were particularly frequently found close to the tight junctions (Figure 6, yellow dashed line labelled in the top left picture). Infection with *C. jejuni* wt revealed apical binding of the bacteria in a time-dependent manner. The wt bacteria bound sequentially over 2 and 4 h, reaching maximal binding at 8 h post infection (Figure 6a). Interestingly, surface-bound wt bacteria were mostly clustered near the cell-to-cell junctions (Figure 6a, orange arrowheads), which in some cases were opened locally to form “intercellular clefts” (Figure 6a, blue arrowheads). In contrast, the ∆*htrA* mutant bound at similar rates to the apical cell surface but was not specifically located near the junctions (Figure 6b). Treatment of the cells with 50 μg/mL purified wt OMVs also revealed non-specific binding to the apical surface (Figure 6c, yellow arrowheads). However, the induction of “intercellular clefts” as seen by *C. jejuni* wt infection was not observed after infection with the ∆*htrA* mutant, nor after treatment with OMVs.

### 3.5. E-Cadherin was Disrupted by C. jejuni Wt Infection, but Not by Treatment with OMVs or rHtrA

**Figure 7:**
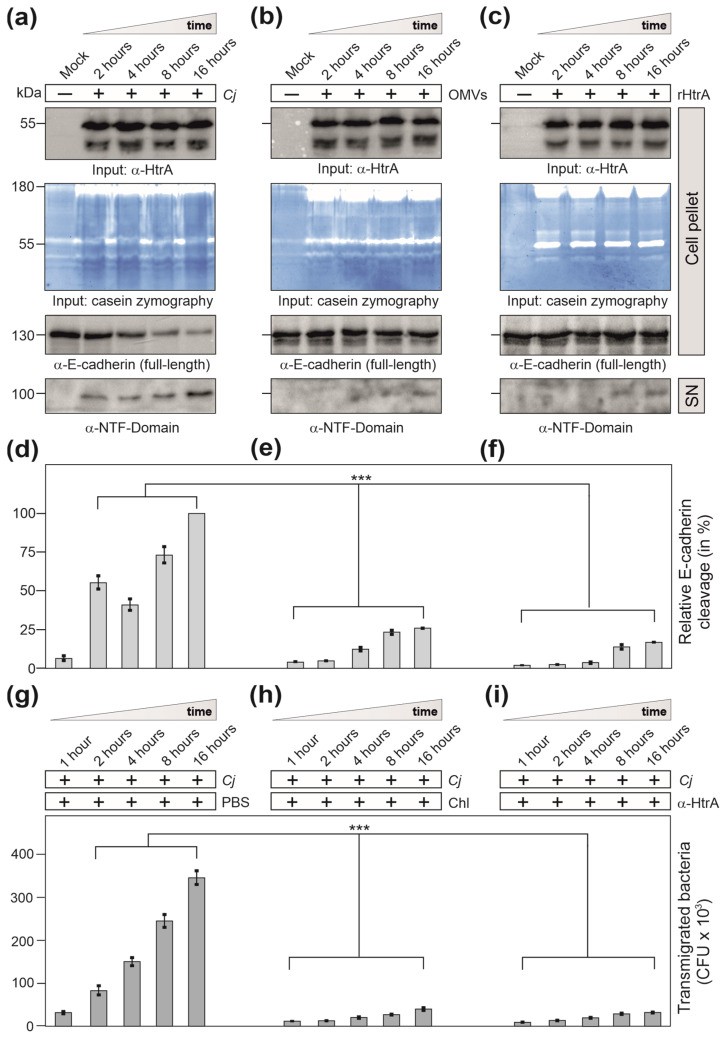
Analysis of E-cadherin disruption in polarized Caco-2 cells during infection with wt *C. jejuni* (**a**) or treatment with wt OMVs (**b**) or rHtrA (**c**). A time course of 2–16 h is shown. Relative E-cadherin cleavage of the extracellular NTF domain after infection with wt *C. jejuni* (**d**) or after treatment with OMV (**e**) or rHtrA (**f**) was quantified from Western blots. The strongest band was set to 100%. (**g**) E-cadherin disruption was associated with enhanced transmigration of wt *C. jejuni* into the basal compartment of the transwell system. Bacterial transmigration was significantly inhibited by addition of chloramphenicol (**h**) or α-HtrA antibodies (**i**). Significant differences shown in the graphs correspond to *p* < 0.001 (***).

In parallel, we analysed E-cadherin cleavage in polarized Caco-2 epithelial cells during infection with wt *C. jejuni* (MOI = 20) or treatment with wt OMVs (50 μg/mL) or rHtrA (1 μg/mL) in a time course of up to 16 h. Western blots and casein zymography confirmed similar amounts of HtrA in each experiment (Figure 7a–c). The HtrA amounts used in the experiments were within the stated physiological conditions, in agreement with a recent study that quantified the secretion of 0.87–0.96 μg/mL HtrA by *C. jejuni* strains 81–176 and NCTC11168 after 8 h of growth [47].

Wt *C. jejuni* infection resulted in profound cleavage of full-length E-cadherin (130 kDa) over time, giving rise to the 100 kDa amino-terminal NTF-fragment released into the supernatant (Figure 7d), but not treatment with wt OMVs (Figure 7e) or rHtrA (Figure 7f). E-cadherin disruption by infection with wt *C. jejuni* was associated with enhanced bacterial transmigration from the apical into the basal compartment of the transwell system (Figure 7g). This suggests that the bacteria triggered their own fast and efficient transmigration through HtrA-dependent cleavage of E-cadherin and other junction proteins, independent of the extracellular release of OMVs or soluble HtrA.

### 3.6. Inhibition of Protein Biosynthesis or Pre-Treatment with Anti-HtrA Antibodies Block C. jejuni Transmigration

Interestingly, bacterial transmigration was strongly inhibited by addition of the protein biosynthesis inhibitor chloramphenicol (Figure 7h) or by pre-treatment of the bacteria for 30 min with α-HtrA antibodies in the apical chamber (Figure 7i). These data indicate that effective *C. jejuni* transmigration requires active bacterial protein biosynthesis and surface-exposure of proteolytically active HtrA, implying that the major proteolytic potential of HtrA maybe present on the bacterial cell surface. In particular, a recent analysis showed that bacterial cell-associated HtrA amounts exceed the signals for secreted HtrA by more than ten-fold [47]. We therefore propose that bacterial cells might expose HtrA onto their surface and exhibit proteolytic activity during direct contact with the host cellular junctions while invading the epithelium.

### 3.7. HtrA-Expressing C. jejuni Promote Bacterial Transmigration

**Figure 8:**
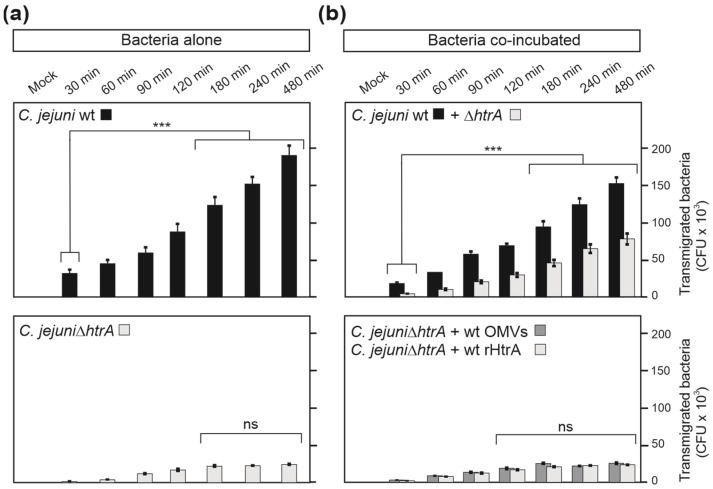
Transmigration properties of *C. jejuni* across polarized Caco-2 cells using a transwell filter system. Caco-2 monolayers grown on transwell filters were infected with either *C. jejuni* wt or Δ*htrA* mutant alone (**a**). Alternatively, the Δ*htrA* mutant was co-incubated with either wt *C. jejuni*, wt OMVs (10 µg/mL)) or wt rHtrA (1 µg/mL) (**b**). Bacterial cells that transmigrated through Caco-2 monolayers into the bottom chambers were collected and quantified on selective agar plates. The experiment was performed in triplicate; ***—*p* ≤ 0.001; ns—non-significant.

To study the role of HtrA in paracellular transport in more detail, we analysed *C. jejuni* transmigration rates during infection with a single strain and during co-infections. In agreement with previous studies [26,37,38], wt *C. jejuni* efficiently transmigrated through the epithelial layer, while deletion of the *htrA* gene significantly reduced the transmigration frequency (Figure 8a). Co-incubation of the Δ*htrA* mutant with wt bacteria efficiently rescued the transmigration deficiency of the mutant (Figure 8b). In contrast, co-incubation of the Δ*htrA* mutant with wt OMVs or wt rHtrA did not increase the transmigration capacity of the mutant. These data confirm our previous statement that the bacteria-associated HtrA likely plays a major role in *C. jejuni* paracellular transport across polarized epithelia, but not shed OMVs or secreted soluble HtrA.

## 4. Discussion

**Figure 9:**
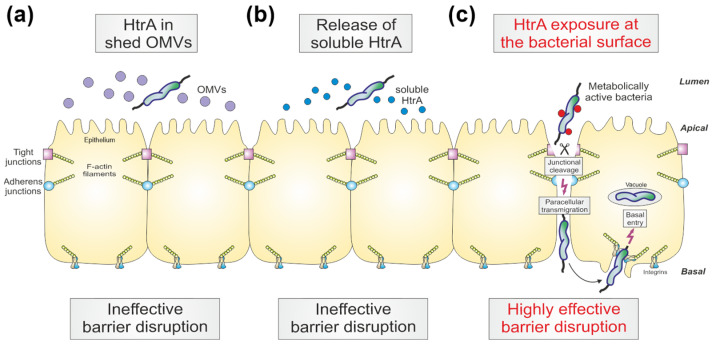
Model of HtrA-dependent disruption of the epithelial barrier during *C. jejuni* infection. There are various options (**a**–**c**). HtrA presentation in the host cellular junctions and induction of epithelial barrier disruption is triggered by shedding of OMVs (**a**), by secretion of soluble HtrA (**b**) or by HtrA exposure at the bacterial cell surface (**c**). Secretion of HtrA-containing OMVs or soluble protein results in sparse damage to cellular junctions, while HtrA exposed at the bacterial surface provides efficient disruption of cellular junctions.

Complex interactions with various hosts are a hallmark of *C. jejuni* biology, which in certain cases lead to the development of pathogenic processes [59,60]. *C. jejuni* has a remarkable potential to infect a variety of different hosts, including humans, cattle, sheep, pigs, poultry, wild birds, and domestic cats and dogs, and has even been isolated from surface water [11,18]. Such a wide host range requires appropriate resources for adaptation to changing environments. Bacterial proteases contribute considerably to both virulence and stress tolerance during host–pathogen interactions [11,28,29,61]. We previously showed that serine protease HtrA plays an important role in heat and oxygen shock tolerance of *C. jejuni*, as well as in host cell adhesion, invasion, and transmigration [26,30,33,34,35,37,38,39,40,41]. The role of HtrA action during the infection process was emerging; however, the exact mechanisms of HtrA delivery remained elusive. The proposed key routes for HtrA-host interaction include (i) secretion of soluble HtrA, (ii) secretion of HtrA within OMVs, and (iii) presentation of membrane-anchored HtrA [27,28,46,47]. Here, we aimed to revise our current understanding of *C. jejuni* HtrA delivery to host cells and propose that surface-associated HtrA is the major pathogen–host interaction route (model in Figure 9).

Using fluorescence microscopy, we observed no or only weak proteolytic cleavage of cellular junctions by either *C. jejuni* OMVs or soluble HtrA. In contrast, infection of epithelia with live *C. jejuni* bacteria resulted in profound damage to epithelial junctions [26,40,41]. The amount of HtrA that can potentially be secreted by *C. jejuni* at a given time varies roughly from 10 ng/mL to 1 μg/mL, while the cell-associated HtrA exceeds these amounts by at least 10 times [46,47]. However, we observed only poor activity of OMVs and rHtrA on cell-to-cell junctions, probably because OMVs and HtrA are not secreted at the appropriate position (Figure 9a,b). Instead, we propose that membrane-bound HtrA on the bacterial surface facilitates and mediates targeted damage of cellular junctions directly at the specific location of bacterial transmigration (Figure 9c). This strategy would be the most effective way to save bacterial energy, applying the protease only when and where it is necessary for *C. jejuni*–host interactions. Our data suggest that surface-exposed HtrA is membrane-anchored by *C. jejuni* bacteria and is only poorly associated with shed OMVs. The proteolytically active domain of membrane-anchored HtrA could undergo the appropriate folding when encountering host cell target molecules, e.g., occludin or claudin-8 in the tight junctions [26,40,41]. The membrane anchoring itself could also provide HtrA with the correct folding when closely attached/integrated with the bacterial cell surface. The analysis of such three-dimensional/conformational changes should be the topic of future experiments.

Our data are in good agreement with previous biochemical fractionation studies on a related pathogen, *H. pylori*, in which HtrA was predominantly found in the structure-bound membrane fraction [62]. Recently, we identified surface-associated HtrA on *H. pylori* via immunostaining and fluorescence microscopy of non-permeabilized bacterial cells [55]. We suggest that *C. jejuni* might have similar phenotypic and functional features of surface-exposed HtrA. The development of antibodies for immunofluorescence microscopy of *C. jejuni* HtrA would help to clarify whether the protease is surface-exposed, as it is on *H. pylori*. Furthermore, wt *H. pylori* showed a tropism towards host cellular junctions, while the Δ*htrA* mutant clustered significantly less at cell junctions [55], providing more evidence for a targeted strategy of HtrA-dependent cleavage of cell junction proteins by either *C. jejuni* or *H. pylori*. In addition, our results correlate with data obtained from purified OMVs of *H. pylori* that contain the translocated effector protein CagA [50,63]. Similar to HtrA from *C. jejuni*, CagA was not translocated and phosphorylated upon co-incubation of OMVs with host cells (according to our unpublished data).

During *C. jejuni* infection, protease HtrA targets the junctional proteins occludin, claudin-8 and E-cadherin, which leads to increased epithelial permeability associated with IBD. Indeed, deregulated expression of junctional proteins is a hallmark in IBD patients [64,65]. In particular, claudin-8 was found to be localized in the cell cytoplasm of intestinal epithelia near mucosal erosions and ulcers [64]. Moreover, both occludin and claudin-8 were shown to be downregulated and redistributed from the cellular junctions in patients with active Crohn’s disease [65]. We recently observed a similar redistribution of occludin and claudin-8 from junctions into the cytoplasm in *C. jejuni*-infected intestinal epithelial cells [41]. Therefore, we and others proposed that commensal microbiota might co-transmigrate along with *C. jejuni*, which in turn can lead to aggravated inflammation and development of related gut disorders [21,22,26]. On the other hand, claudins may play a role beyond tight junctions, e.g., via control of intracellular signalling [64]. It is, however, still unclear whether *C. jejuni* HtrA modulates claudin-8 function towards intracellular signalling activity. Future studies should address this question.

Overall, our findings provide new valuable insights into the mechanism of HtrA-dependent damage to the intestinal epithelium caused by *C. jejuni*. The exact mechanism of surface exposure, however, is still unknown because HtrA does not exhibit high score predictions for a transmembrane domain or autotransporter functions. Yet, bacterial surface localization of *C. jejuni* HtrA, rather than secretion of soluble HtrA or OMVs, appears to be an energy-saving strategy that allows effective targeting of the host substrate upon direct contact, locally opening the cell junctions and triggering bacterial transmigration.

## Data Availability

The data that support the findings of this study are available from the corresponding author upon request.
